# Analyzing Antibiotic Resistance in Bacteria from Wastewater in Pakistan Using Whole-Genome Sequencing

**DOI:** 10.3390/antibiotics13100937

**Published:** 2024-10-04

**Authors:** Fazal Sattar, Xiao Hu, Anugrah Saxena, Kathy Mou, Huigang Shen, Hazrat Ali, Muhammad Afzal Ghauri, Yasra Sarwar, Aamir Ali, Ganwu Li

**Affiliations:** 1 National Institute for Biotechnology and Genetic Engineering College, Pakistan Institute of Engineering and Applied Sciences (NIBGE-C, PIEAS), Faisalabad 38000, Punjab, Pakistan; fazalsattar1226@gmail.com (F.S.); alibiotechnologist@gmail.com (H.A.); ghauri1961@gmail.com (M.A.G.); yasrasarwar@yahoo.com (Y.S.); 2 Department of Veterinary Diagnostic and Production Animal Medicine, College of Veterinary Medicine, Iowa State University, Ames, IA 50011, USA; xiaohu@iastate.edu (X.H.); anugrah@iastate.edu (A.S.); kmou@iastate.edu (K.M.); hgshen@iastate.edu (H.S.)

**Keywords:** whole-genome sequencing, Pakistan, wastewater, antibiotic resistance, carbapenem, plasmid

## Abstract

**Background:** Wastewater is a major source of Antibiotic-Resistant Bacteria (ARB) and a hotspot for the exchange of Antibiotic-Resistant Genes (ARGs). The occurrence of Carbapenem-Resistant Bacteria (CRB) in wastewater samples is a major public health concern. **Objectives:** This study aimed to analyze Antibiotic resistance in bacteria from wastewater sources in Pakistan. **Methods:** We analyzed 32 bacterial isolates, including 18 *Escherichia coli*, 4 *Klebsiella pneumoniae*, and 10 other bacterial isolates using phenotypic antibiotic susceptibility assay and whole-genome sequencing. This study identified the ARGs, plasmid replicons, and integron genes cassettes in the sequenced isolates. One representative isolate was further sequenced using Illumina and Oxford nanopore sequencing technologies. **Results:** Our findings revealed high resistance to clinically important antibiotics: 91% of isolates were resistant to cefotaxime, 75% to ciprofloxacin, and 62.5% to imipenem, while 31% showed non-susceptibility to gentamicin. All *E. coli* isolates were resistant to cephalosporins, with 72% also resistant to carbapenems. Sequence analysis showed a diverse resistome, including carbapenamases (blaNDM-5, blaOXA-181), ESBLs (blaCTX-M-15, blaTEM), and AmpC-type β-lactamases (blaCMY). Key point mutations noticed in the isolates were pmrB_Y358N (colistin) and ftsI_N337NYRIN, ftsI_I336IKYRI (carbapenem). The *E. coli* isolates had 11 different STs, with ST410 predominating (28%). Notably, the *E. coli* phylogroup A isolate 45EC1, (ST10886) is reported for the first time from wastewater, carrying blaNDM-5, blaCMY-16, and pmrB_Y358N with class 1 integron gene cassette of *dfrA*12-*aadA*2-*qacEΔ1* on a plasmid-borne contig. Other carbapenamase, blaNDM-1 and blaOXA-72, were detected in *K. pneumoniae* 22EB1 and *Acinetobacter baumannii* 51AC1, respectively. The integrons with the gene cassettes encoding antibiotic resistance, and the transport and bacterial mobilization protein, were identified in the sequenced isolates. Ten plasmid replicons were identified, with IncFIB prevalent in 53% of isolates. Combined Illumina and Oxford nanopore sequencing revealed blaNDM-5 on an IncFIA/IncFIC plasmid and is identical to those reported in the USA, Myanmar, and Tanzania. **Conclusions:** These findings highlight the environmental prevalence of high-risk and WHO-priority pathogens with clinically important ARGs, underscoring the need for a One Health approach to mitigate ARB isolates.

## 1. Introduction

The rapid emergence and spread of Antibiotic-Resistant Bacteria (ARB) is a global health concern. Particularly, Carbapenem-Resistant Bacteria (CRB), according to the recent World Health Organization (WHO) report, are an alarming threat to public health worldwide [1]. Resistance against carbapenem, the last-resort antibiotic, in the *Enterobacteriaceae* and *Acinetobacter baumannii* complex, has progressively worsened and led to the development of Multi-Drug Resistance (MDR) phenotypes. This is a major challenge for health institutions around the global due to limited antibiotic choices and high mortality rates [2]. In Pakistan, the over-the-counter availability of antibiotics promotes misuse among humans and animals. The use of antibiotic in food-producing animals as prophylactic and growth-promoting agents is poorly controlled [3]. Pharmaceutical manufacturing and agricultural runoffs also contribute to environmental contamination and the persistence of antibiotic compounds in the environment [4]. Inadequate treatment of antibiotic-laden wastewater facilitates their dispersion into aquatic environments such as streams, canals, and rivers, exerting continuous selection pressure on both pathogenic and non-pathogenic bacterial populations. This pressure promotes the transmission of antibiotic resistance genes via plasmids or other mobile genetic elements (MGEs) among different species and even genera. Consequently, this significantly contributes to the emergence and dissemination of Antibiotic-Resistant Genes (ARGs) in diverse environmental settings [5,6]. ARB can potentially infiltrate into drinking water supplies, especially in urban areas of low-resource countries like Pakistan, as reported in a similar developing country, Bangladesh, where the researcher isolated ARB from drinking water facilities [7]. In such cases, wastewater acts as a crucial vector for the transmission of bacterial pathogens to humans in regions with inefficient water quality management systems. Factors such as larger populations, rapid industrialization, and exposure to climatic changes, as seen in Pakistan, pose serious risks to public health and individuals interacting with these environments [8,9].

Integrons are genetic elements identified to facilitate the horizontal gene transfer between bacteria by incorporating exogenous genes [10]. The genetic structure of an integron is composed of the integron integrase (*IntI*), which is a site-specific recombinase, a recombination site (*attI*), and a promoter (Pc) that directs the transcription of foreign genes. The *IntI* mediates the integration that captures, removes, and shuffles circularized genetic components called gene cassettes. These gene cassettes, consisting of a cassette recombination site (*attC*) and an open reading frame (ORF), can lead to diverse adaptive functions [11]. Integrating new gene cassettes, which are diverse and abundant in the environment, can lead to rapid adaptation and help the bacteria evolve in specific traits. The integrons were first identified due to their significant contribution in the spread of antibiotic resistance genes. Recently, it has been revealed that they can encode gene cassettes, which are of an unknown function but often encompass proteins involved in resistance to disinfectants, heavy metals, cell interactions, and niche adaptation [12]. There are three integron classes based on the amino acid sequences of *IntI* integrases as (*IntI*1, *IntI*2, and *IntI*3) that are known to be responsible for resistance to antibiotics. The class 1 integrons are the most predominant among bacteria and are mostly found in the *Enterobacteriaceae* family as well as other clinically significant Gram-negative bacteria [13].

In this study, we investigated thirty-two bacterial isolates from wastewater sources in Pakistan and determined the phenotypic antibiotic resistance profiles. Whole-genome sequencing was performed to identify the ARGs, point mutations responsible for resistance, MLST, plasmid replicons, and integron gene cassettes. Additionally, Oxford nanopore technology was used to sequence one representative isolate to detect the plasmid and the genetic environment of ARGs using the combined Illumina and nanopore reads for that isolate. Although extensive studies have reported antibiotic resistance in bacterial isolates from environmental water sources globally [14,15,16], in Pakistan the majority of the studies focused on the detection of ARGs using the PCR-based amplification approaches from wastewater bacterial isolates [17,18,19,20]. To the best of our knowledge, there are limited studies published regarding the whole-genome sequencing analysis of wastewater samples [21,22]. There are reports regarding the whole-genome sequencing of CRB, but these are from humans, hospital settings (including surfaces, intensive care units, and sinks), animal sources, and food products—not wastewater sources [23,24,25,26,27,28,29]. However, the characterization of ARB and particularly the CRB isolates from wastewater using whole-genome sequencing analysis in Pakistan needs attention because there is little evidence concerning the diversity, spread, and resistome content of the wastewater-borne bacterial population. This investigation aimed to evaluate the prevalence of ARB and CRB in wastewater samples from Pakistan, to characterize the bacterial isolates and their ARGs profiles using phenotypic assay and whole-genome sequencing. Additionally, the plasmid replicons and integron–intergase gene cassettes were investigated in these sequenced isolates. We explored previously overlooked environmental sources and detected the clinically relevant ARGs in these environmental niches, which pose concerns for public and environmental health.

## 2. Results

### 2.1. Bacterial Isolation and Species Identification

The thirty-two bacterial isolates were preliminary identified on selective CHROMagar™ mSuperCARBA culture media according to the provided instructions. The reddish pink and *uidA* positive isolates were identified as *E. coli* and differentiated from the rest of the 14 isolates. All the thirty-two isolates were whole-genome sequenced on an Illumina Miseq platform with an average coverage of ~70×. The genus and species identities were further confirmed using GAMBIT, fastANI, and BIGSdb identification. The isolates were grouped into 7 different genera and 12 different species. Among them, 18 were *E. coli*, 4 *Klebsiella pneumoniae*, and 1 each for *Klebsiella quasipneumoniae*, *Citrobacter telavivensis*, *Citrobacter portucalensis*, *Enterobacter cloacae*, *Raoultella ornithinolytica*, *Raoultella planticola*, *Acinetobacter baumannii*, *Morganella morganii*, *Serratia ureilytica,* and *Serratia nevei*. The assemblies’ statistics, including specific genome sizes, contig numbers, G + C content, N50 values, ANI (%), and the nearest BIGSdb species identification, are shown in Supplementary File Table S1.

### 2.2. Phylogroups, MLST, Serotypes, and Capsule Types

**Figure 1:**
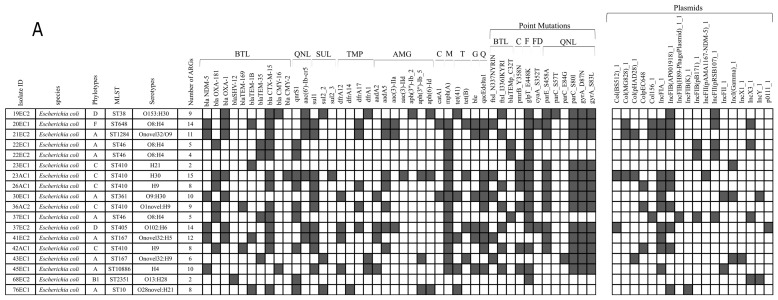

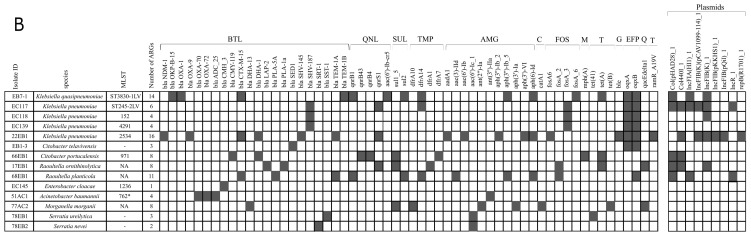
Summary of ARGs detected in the sequenced isolates, (**A**) *E. coli* and (**B**) rest of the isolates. The species column indicates the predicted species for each isolate. The phylotypes and serotypes for *E. coli* were determined along with the MLST for each isolate if the scheme was available. * Pasteur MLST scheme for *Acinetobacter baumannii* 51AC1. “-” in the MLST indicates that the MLST scheme is not available or an unknown MLST. The total ARGs are summarized by bacterial isolate identified *in silico*, and conferring resistance as summarized by antibiotic category. (**A**) Summary of ARGs, point mutations and plasmid replicons in *E. coli* isolates from this study. (**B**) The ARGs in the sequenced isolates are those belonging to *Klebsiella* and other individual species sequenced in this study. BTL, β-lactams; QNL, quinolone; SUL, sulfonamide; TMP, trimethoprim; AMG, aminoglycoside; C, chloramphenicol; FOS, Fosfomycin; M, macrolide; T, tetracycline; G, Glycopeptide; EFP; multi-drug efflux RND transporters; Q; quaternary ammonium compound; T; point mutations for tigecycline. FD, fosmidomycin; Plasmids; the plasmids replicons hits for each isolate.

Classification of the phylogroups of the 18 sequenced *E. coli* isolates was performed and they were grouped into 5 phylogroups (Figure 1A). The most prevalent phylogroup was phylogroup group A (50%; *n* = 9), followed by phylogroup C (28%; *n* = 5), and phylogroup D (11%; *n* = 2). One isolate each for phylogroup B1 and F was detected (Figure 1A).

The MLST analysis revealed diverse sequence types for the studied isolates with some belonging to the pathogenic clonal lineages. The 18 *E. coli* isolates can be grouped into 11 different STs. ST410 was observed as a predominant ST in 5 out of 18 (28%) with all having phylogroup C. ST46 and ST167, both in phylogroup A, were found in 3 and 2 out of 18 isolates (17% and 11%), respectively. The other detected STs belonging to phylogroup A were ST1284, ST361, ST10886, and ST10. The two STs, ST38 and ST405, were the only phylogroup D isolates. The one isolate each for phylogroup B1 and F were ST2351 and ST648, respectively (Figure 1A). The STs reported here, including ST10, ST410, ST167, and ST46, are pathogenic clonal lineages reported globally. ST10, ST167, and ST1284 are classified into Clonal Complex 10 (CC10) according to the EnteroBase, which are the pathogenic lineages [30].

The serotypes were also identified for the *E. coli* isolates. The most frequent ST, ST410, was found to have different serotypes, including H9 (*n* = 2), H21 (*n* = 1), H30 (*n* = 1) and O1novel:H9 (*n* = 1). The STs, including ST648 and ST46, have the same serotype O8:H4 (*n* = 4), while the two ST167 have different serotypes: Onovel31:H5 (*n* = 1) and Onovel32:H9 (*n* = 1). The other detected serotypes with STs included ST38 of serotype O153:H30, ST1284 of serotype Onovel32/O9, ST361 of serotype O9:H30, ST405 of serotype O102:H6, ST10886 of serotype H4, ST2351 of serotype O13:H28, and ST10 of serotype O28novel:H21 (Figure 1A).

For *Klebsiella*, the in silico MLST analysis showed that all four isolates were of different sequence types including ST3830-ILV, ST245-2LV, ST152, ST4291, and ST2534 (Figure 1B). Sequence variations within the capsular polysaccharides, K antigens, and lipopolysaccharides such as O antigens, are another typing method for K. pneumoniae. In the studied isolates, three distinct O capsule types (O1/O2v1, O1/O2v2, and O3b) and four distinct K capsule types (KL62, KL102, KL104 and KL105) were detected (Supplementary File Table S3).

### 2.3. Antimicrobial Sensitivity Assay (ASA)

The ASA analysis against ten antibacterial agents of thirty-two isolates is presented in Table 1. Overall, the isolates displayed resistance against the cephalosporin, i.e., cefotaxime in 31 out of 32 (97%) isolates, followed by ciprofloxacin in 29 out of 32 (91%) isolates. Regarding carbapenem, 25 out of 32 (78%) isolates were resistant to imipenem. The sensitive drugs identified were gentamicin followed by trimethoprim-sulfamethoxazole. The resistance against these drugs were found in 18 (56%) and 20 (63%) of the 32 isolates as shown in Table 1.

In the *E. coli* isolates, 11 out of 18 were resistant to all antibiotics tested. Additionally, two isolates were resistant to nine antibiotics, while resistance against eight, seven, and six antibiotics was found in one isolate, three isolates, and one isolate, respectively (Table 1). Resistance to carbapenems (imipenem, meropenem, doripenem, and ertapenem) was noticed in 16 out of 18 (89%) *E. coli* isolates. Regarding cephalosporins, all isolates (100%) were resistant to cefotaxime and ceftazidime, except for 42AC1, which was sensitive to the latter. Similarly, 42AC1 was the only isolate sensitive to ciprofloxacin and nalidixic acid, while all other *E. coli* isolates were resistant. Among the 18 *E. coli* isolates, 13 (72.2%) were resistant to trimethoprim-sulfamethoxazole and gentamicin. The isolate 42AC1 was the least resistant isolate, followed by 76EC1, being sensitive to eight and four antibiotics, respectively.

In the case of *Klebsiella*, the *Klebsiella quasipneumoniae* EB7-1 displayed non-susceptibility to all antibiotics, followed by *Klebsiella pneumoniae* 22EB1, which was only sensitive to trimethoprim-sulfamethoxazole. The isolate EC118 was resistant to meropenem, cefotaxime, and ciprofloxacin. The *Klebsiella pneumoniae* EC117 was sensitive to ceftazidime and gentamicin, while EC139 was sensitive to imipenem, nalidixic acid, and trimethoprim-sulfamethoxazole in addition to gentamicin (Table 1).

Furthermore, among the nine out of thirty-two isolates with single species, the *Raoultella planticola* 68EB1 isolate was resistant to all antibiotics tested. Of these nine isolates, seven were resistant to carbapenem, especially imipenem and doripenem. All nine isolates were resistant to cefotaxime, except *Raoultella ornithinolytica* 17EB1, which was sensitive to cefotaxime but resistant to ciprofloxacin and trimethoprim-sulfamethoxazole. The *Citrobacter telavivensis* isolate EB1-3 was resistant to meropenem, doripenem, cefotaxime, and ciprofloxacin but sensitive to all other antibiotics. The *Citrobacter portucalensis* isolate 66EB1 was sensitive to meropenem, ciprofloxacin, and gentamicin while the *Enterobacter cloacae* EC145 isolate was sensitive to meropenem, nalidixic acid, and trimethoprim-sulfamethoxazole. (Figure 1). Also, the *Acinetobacter baumannii* 51AC1 isolate was sensitive to nalidixic acid and gentamicin, whereas the *Morganella morganii* 77AC2 was only sensitive to ertapenem. Both the *Serratia* isolates, including *Serratia ureilytica* 78EB1 and *Serratia nevei* 78EB2, were resistant to imipenem, cefotaxime, and ceftazidime; the former was additionally resistant to imipenem and doripenem (Table 1).

### 2.4. Detection of ARGs

The whole-genome sequencing of thirty-two ARB recovered from wastewater displayed a diverse resistome, including the two clinically relevant carbapenamases, NDM and OXA. Other important β-lactamases, including CTX-M-15, TEM, SHV, and CMY genes, were also noticed in some of the isolates (Figure 1A).

In *E. coli* isolates, 15 resistance genes were found in the isolate 23AC, while on average, 8 ARGs were detected in all other isolates. Notably, the critical carbapenamase genes, *bla* _NDM-5_ and *bla* _OXA-181_, were each present in 6 out of 18 (33%) isolates. The six isolates bearing *bla* _NDM-5_ belonged to different STs including ST648, ST1284, ST361, ST405, ST167, and ST10886. On the other hand, *bla* _OXA-181_ was found in two STs, including ST46 (*n* = 2) and ST410 (*n* = 4). Additional β-lactamases were found, including *bla* _CTX-M-15_ in 13 out of 18 (72%) isolates, and *bla* _OXA-1_ in 7 out of 18 (38%) isolates. Variants of the *bla* _TEM_ and *bla* _CMY_ genes were present in 10 out of 18 (56%) and 3 out of 18 (17%) isolates, respectively, while *bla* _SHV-12_ was identified in only one isolate, 68EC2. Regarding quinolone resistance, genes such as *qnrS1* and *aac(6′)-Ib-cr* (which confer resistance to both quinolones and aminoglycosides) were found in 14 out of 18 (77.7%) isolates, and isolate 23AC1 co-harboring both the genes. Sulfonamide and trimethoprim resistance genes (*sul* and *dfrA*) were detected in 13 and 11 out of 32 (72% and 61%) isolates, respectively. Isolates 23AC1 and 37EC2 co-harbored two genes each for sulfonamide (*sul*1 and *sul*2) and trimethoprim (*dfrA*12 and *dfrA*17) on different contigs in their genomes. On the other hand, the isolate 45EC1 contained two *dfrA* genes (*dfrA*1 and *dfrA*12) on separate contigs. Aminoglycosides-resistant genes were detected in 13 out of 18 (72%) isolates, with 10 of these harboring the variants of *aadA* including the *aadA*5 and *aadA*2 genes. Interestingly, the isolate 37EC2 encoded both the *aadA*5 and *aadA*2 genes. Other important ARGs, for which phenotypic susceptibility was not tested, included the macrolide resistance gene (*mph(A)*) in 15 out of 18 (83%) isolates, the chloramphenicol resistance gene (c*atA*1) in 2 out of 18 (11%) isolates, and the tetracycline resistance genes (*tet(A)* and *tet(B)*) in 9 out of 18 (50%) isolates. The glycopeptide-resistant gene, *ble*, was found in all six isolates harboring the *bla* _NDM-5_ gene. Additionally, the *qacEdelta1* gene associated with quaternary ammonium compound efflux pump transporter, was present in 10 out of 18 (55%) isolates.

Point mutations conferring antibiotic resistance were also detected in the sequenced *E. coli* isolates. Notably, the *pmrB* (Y358N) mutation was detected in 6 out of 18 (33%) isolates, known for heteroresistance to colistin. Additionally, 2 chromosomal point mutations conferring resistance to carbapenems were identified in 12 out of 18 (67%) *E. coli* isolates, including five isolates with the ftsI_N337NYRIN point mutation and seven isolates with the ftsI_I336IKYRI. The glpT_E448K point mutation for fosfomycin resistance was noticed in 15 out of 32 (83%) *E. coli* isolates. Furthermore, the mutations, blaTEMp_C32T and cyanA_S352T (fosmidomycin resistance) were detected in 3 and 2 out of 18 (17% and 11%) isolates. Mutations in the *gyrA*, *parC*, and *parE* genes responsible for quinolone resistance were also detected in the *E. coli* isolates. More specifically, the amino acid substitutions, S83L and D837N in *gyrA* and S80I in the *parC* gene, were detected in 13 out of 18 (72%) isolates. The S458A mutation in the *parE* gene was present in 11 of 18 (61%) isolates. Additionally, the S57T and E84G in the *parC* genes were only detected in one isolate each, as shown in Figure 1A.

Regarding *Klebsiella*, the *Klebsiella quasipneumoniae*, EB7-1 isolate harbored 14 ARGs, including the β-lactamases *bla* _OKP-B-15,_ *bla* _OXA-1,_ *bla* _CTX-M-15,_ and *bla* _TEM-1B_. Other detected ARGs included the *qnrB1*, *aac(6′)-Ib-cr*5, *sul*2, *dfrA*14, *aph(3″)-Ib*, *aph(6)-Id*, *fosA_6*, and *tet(A)*. Additionally, isolates were found to harbor genes encoding multi-drug efflux RND (Resistance-Nodulation-Division) transporter periplasmic adaptor subunit (*oqxA*) and multi-drug efflux RND transporter permease subunit (*oqxB*). The isolate EC117 included the *bla* _SHV-187_, *qnrS1*, *oqxA/B*, *dfrA*14, and *fosA_3*. The *Klebsiella pneumoniae* strains EC118 and EC139 harbored the same ARGs, including *bla* _SHV-187_, *oqxA/B*, and *fosA_3*. Among the *Klebsiella* isolates, the *Klebsiella pneumoniae* 22EB1 isolate carried 16 ARGs including β-lactamases such as *bla* _NDM-1_, *bla* _OXA-9_, *bla* _CTX-M-15_, *bla* _DHA-1_, *bla* _SHV-145_, and *bla* _TEM-1A_. This isolate also harbored additional ARGs such as *qnrS1*, *oqxA/B*, *aadA*1, *aac(6′)-Ib*, *aph(3″)-Ib*, *aph(3′)-VI*, *aph(6)-Id*, *ble*, and *fosA_3* (Figure 1B).

Additionally, amino acid substitutions for tigecycline resistance were detected in the carbapenem-resistant *Klebsiella pneumoniae* 22EB1 isolate. The mutation in the *ramR* gene (A19V), which is part of the *AcrAB* efflux pump, was found in the isolate. However, this has previously been identified to have no significant role in the tigecycline resistance [31].

Moreover, in nine out of the thirty-two isolates from single species, the *Citrobacter telavivensis* strains EB1-3 harbored *bla* _SED1_ and *oqxA/B*, while *Citrobacter portucalensis* 66EB1 was found to have *bla* _CMY-119_, *bla* _DHA-1_, *qnrB43*, *qnrB4*, *sul1*, *dfrA7*, *mph(A)*, and *tet(A)*. The *Raoultella ornithinolytica* strains 17EB1 harbored *bla* _LAP-2_, *bla* _PLA-1a_, *qnrS1*, *sul1*, *dfrA1*, *fosA_2*, and *tet(B),* while *Raoultella planticola* 68EB1 was found to have *bla* _CTX-M-15_, *bla* _PLA-5A_, *bla* _TEM-1A_, *qnrB1*, *oqxB sul2*, *dfrA14*, *aac(3)-IId*, *aph(3″)-Ib*, *aph(6)-Id*, and *fosA_2*. The *Enterobacter cloacae* EC145 harbored one gene of *bla* _CMH_3_ while the *Acinetobacter baumannii* 51AC1 harbored *bla* _OXA-70_, *bla* _OXA-72_, *bla* _ADC_25_, and *ant(3”)_IIa*. In *Morganella morganii* 77AC2, *bla* _DHA-13_, *sul1*, *dfrA10*, *ant(2”)_Ia*, *aph(3′)-Ia*, *catA1*, *tet(B)*, and *qacEdelta1* were detected. The two *Serratia* harbored *aac(6′)-Ic*, and the *bla* _SST-1_ and *bla* _SRT-1_ in *Serratia ureilytica* and *Serratia nevei*, respectively, as shown in Figure 1B. Most of the isolates confirmed the genotypes for a specific phenotype, and a correlation of the genetic resistance mechanism responsible for a phenotype is provided in Table 2.

### 2.5. Detection of Integrons–Integrases

The isolates from this study were investigated for the presence of integrons, the genetic elements identified for the spread of gene cassettes conferring adaptive functions. Table 3 summarizes the prevalence of integrons and their gene cassettes carrying different genes in the integron-positive isolates. Of the 32 isolates sequenced from this study, 11 harbored a class 1 integron integrase gene. Among these 11, 6 were *E. coli*, accounting for 55% of the total, while others included *Klebsiella quasipneumoniae*, *Klebsiella pneumoniae*, *Citrobacter portucalensis*, *Raoultella planticola*, and *Morganella morganii*. The integron was present on the plasmid-borne contig of all the isolates except for *Citrobacter portucalensis*, where the integron was found on a chromosomal-borne contig. All the *E. coli* isolates carried the same array of genes in the gene cassettes: genes encoding resistance to trimethoprim (*dfrA*), aminoglycosides (*aadA*), and a semi-functional derivative of a small multi-drug resistance (SMR) protein with a role in biocide resistance (*qacEΔ1*). The one exception was for 76EC1, which only contained the *dfrA* gene and a partial IS6 family transposon. The *E. coli* isolates were found to have three and two variants of the *dfrA* and the *aadA* gene, respectively, along with the common *qacEΔ1* gene. Among the six *E. coli* integron-positive isolates, five co-harbor *bla* _NDM-1_ while 76EC1 did not (Figure 1A). The integron-positive isolates from the *Klebsiella* genus, including *Klebsiella quasipneumoniae* and *Klebsiella pneumoniae*, were found with the same array of genes: the trimethoprim resistance gene *drfA* and the bacterial mobilization protein, *mobC*. The integron with the gene cassette array containing *dfrA7* and *qacEΔ1*was found on the chromosomal contig of a *Citrobacter portucalensis*. Interestingly, the *Raoultella planticola* was the only isolate with only the *drfA14* gene in an integron cassette on a plasmid. The *Morganella morganii* isolate 77AC2 was found to carry an aminoglycoside gene, *ant(2′)-ia* (“2”-aminoglycoside nucleotidyltransferase), along with the *qacEΔ1* gene (Table 3).

### 2.6. Detection of Plasmid Replicons

The PlasmidFinder was used to predict the plasmid replicons in the sequenced isolates. The replicons were grouped into ten types: Col, IncFIA, IncFIB, IncFII, IncI, IncR, IncX, IncY, repB, and p0111. IncFIB was the major replicon detected, found in 17 out of 32 (53%) isolates, followed by Col, IncFIA, and IncFII in 15, 13, and 8 (46%, 40%, and 25%) isolates, respectively.

The IncFIB replicon was also detected in most of the *E. coli* isolates (12 out of 18 (67%)), followed by IncFIA in 10 out of 18 (56%) isolates. Six replicons were detected in both 23AC1 and 20EC1; in contrast, only one replicon was detected in 23EC1 and 41EC2 (Figure 1A).

In *Klebsiella*, IncFIB was detected in all isolates except EC139 (no replicon was detected). *Klebsiella quasipneumoniae* EB7-1 was found to have three replicons (Col, IncFIB, and IncFIA). There were six replicons in 22EB1 and EC117, while the isolate EC118 had only one replicon (Figure 1B).

Moreover, among the 9 out of 32 isolates with single species, there were 3 replicons in *Raoultella ornithinolytica* and *Raoultella planticola* isolates. The *Citrobacter portucalensis* 66EB1 isolate was found to have two Col-type plasmid replicons. No plasmid replicons were predicted by the PlasmidFinder tool in *Enterobacter cloacae*, *Citrobacter telavivensis*, *Morganella*, and the two *Serratia* isolates (Figure 1B).

### 2.7. Plasmid Sequence Analysis of E. coli 41EC2 and Localization of Its ARGs

**Figure 2:**
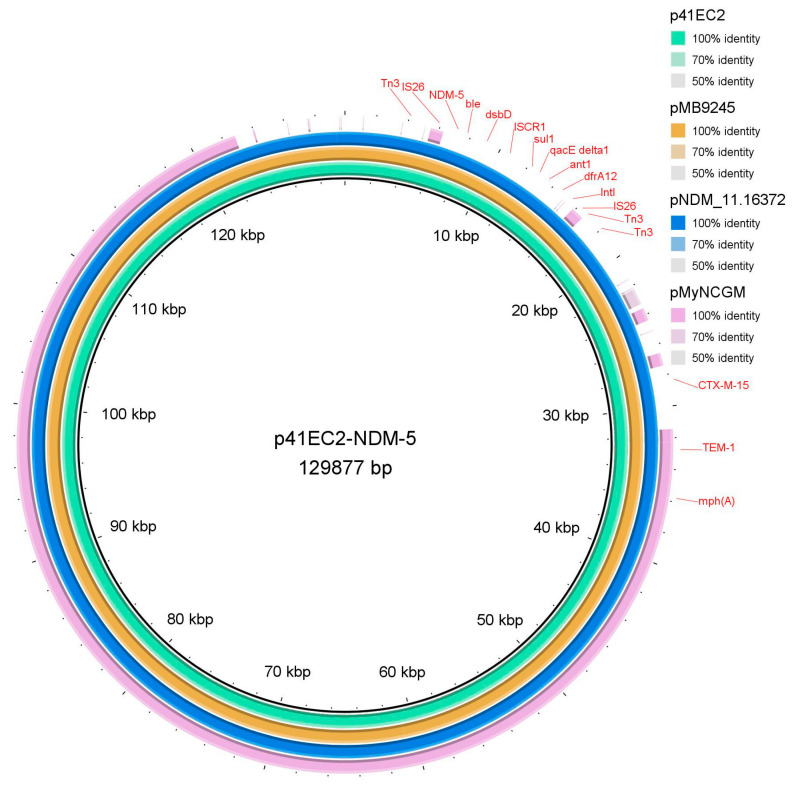
Comparison map of p41EC2 with other *E. coli* plasmids from NCBI.

To further investigate the plasmid content, genetic environment, and localization of the bla NDM-5 gene in the sequenced *E. coli* isolates, a representative 41EC2 isolate was further selected. The combined long and short read sequencing approach was utilized to obtain a full-length complete plasmid sequence from the isolate 41EC2. The combined Illumina and nanopore reads for 41EC2 resulted in a single plasmid, identified as IncFIA/IncFIC/rep_cluster_2244 (p41EC2; GenBank accession: CP155728) with a size of 129,877 bp. Plasmid multilocus sequence typing (pMLST) using the pMLST tool (https://cge.food.dtu.dk/services/pMLST/) (accessed on 19 July 2024) identified that the p41EC2 belongs to the IncF F36:A4:B-subtype. The p41EC2 was identified as harboring a total of 13 resistance genes (Figure 2). These included 1 biocide and 12 acquired ARGs. Particularly, the carbapenamase-encoding *bla* _NDM-5_, which contributes to the nonsusceptibility phenotype to the last-resort antibiotic, carbapenem, was recorded in this strain, as shown in Table 1 and Figure 1. Other important β-lactamases, the *bla* _OXA-1_, *bla* _CTX-M-15_, and *bla* _TEM-1_, along with other ARGs, such as *tet(A)*, *mph(A)*, *dfrA12*, *sul1*, *aac(6)-Ib-cr1*, *aadA2*, *ble*, and *catB3* (truncated) by *IS*26 element, were also found on the same plasmid. The p41EC2 encompasses a variety of IS elements, including those belonging to the *IS*1 and *IS*6 families. Within this plasmid, blaNDM-5 and numerous ARGs in the center were surrounded by two insertion sequences, *IS*26, which comprises *intI*1 (class 1 integron integrase), and *ISCR1* (insertion sequence common region 1) (Figure 2).

## 3. Discussion

The circulation of Gram-negative ARB in the environment in Pakistan remains poorly understood due to limited data availability. Among ARB, CRB are of concern, having been designated as critical-priority pathogens by the WHO [32]. While most reports of CRB in this region are from clinical settings, their presence in environmental matrices is a growing global concern, with evidence of their spread in community settings, sewage environments, and wastewater sources [33]. The dissemination of Gram-negative ARB isolates into environmental sources poses a serious public health threat. This study documents the presence of clinically relevant ARGs in 32 sequenced bacterial isolates from various sites in Pakistan, including household water, sewage, and canals. These findings suggest the community-level carriage, disposal, and spread of ARB into the environment. The bacterial species were identified using the *k-mer*-based identification tool, followed by ANI analysis and BIGSdb [34]. The isolates comprised *18 Escherichia coli*, 4 *Klebsiella pneumoniae* and 10 other bacterial species. The phenotypic assay revealed a high resistance rate to cephalosporins, ciprofloxacin, and imipenem with most *E. coli* isolates showing resistance to carbapenem. These findings are consistent with previous studies on wastewater isolates from Pakistan, which reported high levels of resistance to β-lactams and fluoroquinolones, including cephalosporin, carbapenem, and ciprofloxacin [18,22]. Our results align with those earlier studies, providing further evidence of the escalating burden of antimicrobial resistance in the South Asian region[35,36].

This project also highlights the presence of diverse sequence types (ST) among the bacterial isolates carrying important ARGs, particularly the carbapenemases. Notably, *bla* _OXA-181_ and *bla* _NDM-5_ were prevalent among the *E. coli* isolates in this study. The *bla* _OXA-181_ gene was found in two STs, the ST361 and ST46, which to our knowledge, are reported for the first time in wastewater isolates from Pakistan. Previous studies have reported the *bla* _NDM-5_ gene in *E. coli* from human and black kites (*Milvus migrans*) in the same region, with a wide range of STs, including ST167, ST361, ST648, and ST1284 [37,38,39]. Our study extended these findings by identifying these high-risk, human-associated *E. coli* clones in wastewater isolates. Importantly, this study reports the detection of a rare *E. coli* ST10886 isolate, 45EC1, belonging to phylogroup A with serotype H9. This isolate harbor the *bla* _NDM-5_ and *bla* _CMY-16_ genes, along with a *pmrB* (Y358N) mutation conferring resistance to colistin (Figure 1). According to the *Escherichia/Shigella Enterobase* database (https://enterobase.warwick.ac.uk) (accessed on 14 June 2024), only three isolates of this ST have been previously documented. Given the challenges posed by poor sanitation and limited access to clean water, the close monitoring of this ST for the *bla* _NDM-5_ gene is crucial, as it may spread through human food chain and emerge as a high-risk, carbapenem- and colistin-resistant *E. coli* clone. Additionally, the *bla* _NDM-1_ gene was detected in the *Klebsiella pneumonia* ST2534 isolate (22EB1), which also harbors the *bla* _DHA-1_ and *bla* _OXA-9_ genes. This study reports this ST for the *K. pneumonia* for first time from this region. Similar findings have been observed in *Klebsiella pneumonia* ST437 and ST101 clones carrying the *bla* _NDM-1_, *bla* _CTX-M-15_, and *bla* _DHA-1_ detected in a patient from Pakistan who was hospitalized in Spain [40]. The *bla* _NDM-1_ gene has been widely reported in *Klebsiella pneumonia* from various human and animal sources in the same region [41,42,43]. The BLASTn analysis revealed that this gene is closely related to *bla* _NDM-1_ from different isolates globally, with the highest similarity to a *Providencia vermicola* human isolate from Nepal (Supplementary File Table S4). The detection of these variants underscores the widespread distribution of *bla* _NDM_ gene variants in wastewater isolates. Furthermore, the *Acinetobacter baumannii* 51AC1 isolate was found to be resistant to carbapenem due to the presence of the *bla* _OXA-72_ gene, in addition to the *bla* _OXA-70_ gene (intrinsic in *Acinetobacter baumannii*) (Table 2). This finding aligns with previous reports on *Acinetobacter baumannii* isolates from clinical samples in this region [44]. The phenotypic and genotypic carbapenem resistances were consistent for most of the sequenced isolates in this study except for three *E. coli* isolates, 23EC1, 68EC2, and 76EC1 (Table 2). Additionally, *Klebsiella quasipneumoniae* EB7-1 was resistant to four carbapenem antibiotics, while the *Morganella morganii* 77AC2 and *Serratia ureilytica* 78EB1 isolates, which were non-susceptible to at least two carbapenems, did not show the genetic evidence of carbapenem resistance. Carbapenem resistance is a complex phenomenon, and beyond acquired genes, these isolates could be utilizing other mechanisms such as novel enzymes, efflux pumps, or changes in the membrane permeability to mediate resistance.

Additionally, Extended Spectrum β-lactamases (ESBLs), including the Cefotaximase-Munich (CTX-M), Sulfhydryl Variable (SHV), and Temoneria (TEM) families, were identified in the studied isolates. Our results showed that CTX-M-15 was the most prevalent ESBL, present in 72% (13 out of 18) of the *E. coli* isolates. Notably, 10 of these 13 isolates co-harbored carbapenemases, specifically the *bla* _NDM-5_ and *bla* _OXA-181_. The high prevalence of ESBLs, particularly CTM-15, has been consistently reported in wastewater samples from this region [17].

Additionally, resistance to β-lactam was also observed due to the presence of AmpC types β-lactamases. AmpC β-lactamases are important cephalosporinases encoded either chromosomally (intrinsic) or by plasmids (acquired) [45]. In this study, the plasmid mediated AmpC β-lactamase *bla* _CMY_, which was previously reported as the first plasmid mediated AmpC β-lactamase [46], was only detected in three *E. coli* isolates and one *Citrobacter portucalensis* isolate (66EB1). Other types of AmpC β-lactamases identified in the sequenced isolates included the *bla* _OKP-B-15_ gene, a chromosomal β-lactamase gene found in *Klebsiella quasipneumoniae*, the *bla* _CMH-3_ gene in *Enterobactor cloaceae*, *ADC* (Acinetobacter-derived cephalosporinases) in *Acinetobacter baumannii*, and *bla* _DHA-1_ in *Klebsiella quasipneumoniae*, *Citrobacter portucalensis* 66EB1, and *Morganella morganii*. Furthermore, the *bla* _PLA-1a_ and *bla* _PLA-5a_ genes, identified as class A β-lactamase, were present in *Raoultella* species. These genes have previously been reported as part of the *Raoultella* chromosome, providing the bacteria with intrinsic resistance to β-lactamases [47]. Another class A β-lactamase, *bla* _LAP-2__, _known for its narrow resistance spectrum against β-lactam antibiotics, was identified alongside the *qnrS1* gene in *Raoultella ornithinolytica* 17EB1 on a single contig. Previously, a similar gene arrangement was found in Col- and IncFIB-type plasmids in *Klebsiella pneumoniae* [48]. The presence of Col- and IncFIB-type replicons in these isolates suggests a similar backbone structure to that found in *Klebsiella pneumonia*, now reported in *Raoultella ornithinolytica* from Pakistan.

The phenotypic resistance to quinolones was observed in most isolates, with the exception of 42AC1 and a few others. This resistance was primarily due to acquired genes such as *qnrS1*, *aac(6)-Ib-cr*, *qnrB1*, *qnrB43* and *qnrB4*, along with multi-drug efflux proteins *oqxA* and *oqxB*, particularly in *Klebsiella* and one *Raoultella* species. Additionally, mutations in quinolone resistance-determining regions (QRDRs) were detected in over 72% of *E. coli* isolates, specifically the amino acid substitutions D87N, S83L, and S458A in DNA *gyrase* (*gyr*A) and topoisomerase IV (*parE*). These substitutions have been strongly associated with high levels of quinolone resistance in *E. coli*, consistent with previous reports [49]. Interestingly, although the *E. coli* isolate 41AC1 carried genetic markers for quinolone resistance, it may be non-susceptible to other quinolones, such as levofloxacin, norfloxacin, and ofloxacin, which were not included in the phenotypic sensitivity tests conducted in this study.

Aminoglycoside resistance genes were in 72% of the *E. coli* isolates, with the *aadA5* gene being most prevalent. Notably, some *E. coli* isolates (42AC1, 19EC2, and 76EC1) possessed one or more aminoglycoside-modifying enzymes (AMEs) that did not correspond with the phenotypic resistance patterns observed (Table 2). This discrepancy suggests potential resistance to additional aminoglycosides, such as amikacin, streptomycin, kanamycin, and tobramycin, which were not tested in the phenotypic assays conducted in this study.

Additionally, genes conferring resistance to macrolide, fosfomycin and tetracyclines were also identified in the studied isolates. These findings are consistent with previous reports from the same region, which detected similar resistance genes in different bacterial isolates [50]. Interestingly, in our study, the *fosA* gene was exclusively found in the *Klebsiella* and *Raoultella* species. It may be attributed to the presence of the *fosA* gene in the chromosome, which confers intrinsic resistance to Fosfomycin and occurrence that is rarely identified in *E. coli* [51].

Previously, colistin resistance due to the *mcr-1* gene has been reported in Pakistan in *E. coli* isolates originating from humans, wild migratory birds, healthy broiler chickens [52], and sewage samples [20]. The later study highlighted a 35% resistance rate to colistin in *E. coli*, underscoring the significant burden of colistin resistance in environmental settings compared to clinical contexts, which is a serious concern. In our study, we observed a mutation in the *pmrB* gene (Y358N) in 33% of *E. coli* isolates. This nucleotide substitution is noteworthy as it has been previously associated with colistin non-susceptibility, exhibiting a high MIC > 32 μg/mL even in the absence of the *mcr* gene [53]. This suggests that the single amino acid change could contribute to colistin resistance. Given these isolates were derived from wastewater, the presence of colistin in such environments may have driven the emergence of resistance. The potential for these resistant isolates to spread to other sources, such as community water supplies, poses a significant public health risk. Therefore, the detection of this mutation in wastewater-borne *E. coli* is concerning and underscores the need for ongoing surveillance and proactive measures to prevent the dissemination of resistant strains.

The identified plasmid replicons indicate that most of the *E. coli* and *Klebsiella* isolates were prone to plasmid uptake, which may facilitate horizontal gene transfer and contribute to the dissemination of critical ARGs among pathogenic bacteria. The *bla* _NDM-5_ isolates exhibited a combination of diverse plasmid replicons, including Col, IncFIA, IncFIB and IncFII types. These replicon types have previously been associated with *bla* _NDM-5_ genes from clinical samples [37]. Additionally, our study also identified IncX3 replicons in two isolates carrying the *bla* _OXA-181_ gene. These findings are consistent with the previous observation linking InX3 replicons to the *bla* _OXA-181_ gene in *Enterobacterales* [54].

The *bla* _NDM-1_ gene in *Klebsiella pneumoniae* 22EB1 ST2534 is likely carried on an IncFIB(pQil) replicon-type plasmid. Similarly, the ESBL gene in this same isolate is probably located on a repB(R1701) replicon-type plasmid. These replicon-type plasmids have been recently identified in clinical isolates associated with the ESBL antibiotic group in Pakistan [43]. In contrast, there were no plasmid replicons detected in the *Acinetobacter baumannii* 51AC1 isolate, which harbors the carbapenamase *bla* _OXA-72_. This could be because the novel replicon type is not present in the database, was missed due to short read sequencing, and/or was present on the chromosome, all of which merit further investigation. 

The representative *bla* _NDM-5_-positive *E. coli* isolate 41EC2 was additionally sequenced by using both Illumina and Oxford nanopore platforms to assemble the full-length plasmid sequence. Combined Illumina and nanopore sequencing reads revealed a single conjugative plasmid in the *E. coli* isolate 41EC2. BLASTn analysis identified this plasmid as being closely related to plasmids in the NCBI database, with 100% coverage and nucleotide identity to other ST167 *E. coli* isolates from USA, including the pMB9245_1 plasmid, GenBank accession No. CP103530.1, with a size of 133,836 bp. Other closely related plasmids included pMyNCGM167_1 (GenBank accession No. LC744455.1, 132,545 bp from Myanmar), pM309-NDM5 (GenBank accession No. AP018833.1, 136,947 bp from Myanmar), and a plasmid from the *E. coli* strain NDM_11.16372 (GenBank accession No. CP082130.1, 136,961 bp from Tanzania), with 100% coverage and 99.99% nucleotide identities, respectively. Concentric ring-based circular comparisons between p41EC2 and similar plasmids (Figure 2) demonstrated that all these plasmids share the IncFIA/IncFIC/InFII-type replicon, consistent with the IncFIA/IncFIC/rep_cluster_2244 type of p41EC2. Previous reports have described similar IncF-type plasmids where the *bla* _NDM-5_ is flanked by two copies of *IS*26, and with *ISCR1* located in between [37,55]. These results highlight the critical roles of *IS*26 and *ISCR1* in the transmission of *bla* _NDM-5_ and underscore their significance in the dissemination of antibiotic-resistant elements. The presence of *bla* _NDM-5_ in this conjugative plasmid likely facilities its spread. The identification of similar plasmids in isolates from various countries suggests a global distribution of such plasmid types.

Furthermore, the intergon–integrase elements were identified in the sequenced isolates. The class 1 integron was found in 55% of the isolates from this study, which is reported for the first time from the wastewater-borne sequenced isolates from Pakistan. Previous studies reported similar percentages of class 1 integrons in clinical isolates [56]. The integrons found in the *E. coli* isolates shared the same genetic array as those present in a clinical class 1 integron [12]. The presence of *qacEΔ1* at the 3′ conserved region suggests it is a clinical class 1 integron that has made its way into the wastewater. Interestingly, this study identified the presence of the *mobC* gene in the integron cassette. The *mobC* is a plasmid-encoded protein making up the relaxosome and assists in the conjugative transfer of plasmids [57]. The integrons present in plasmid-borne contig indicate the possibility of disseminating these genes cassettes into different pathogenic and non-pathogenic bacterial isolates in the wastewater system. The co-occurrence of integrons and the *bla* _NDM-5_ gene in *E. coli* isolates underscores the ability of the genomes to adapt and evolve in response to the environmental changes and conditions. The integrons present in these isolates offer a probable route of transmitting these gene cassettes or transmitting them into a new array with other important resistance genes. This may pose a serious threat to drinking water facilities, such as those in Pakistan with limited wastewater treatment options.

This study highlights the detection of clinically relevant ARGs in bacterial isolates from wastewater, emphasizing the need for a One Health-oriented molecular surveillance approach to combat the growing threats of antibiotic resistance. The numerous reservoirs and dissemination routes of AGRs suggest the importance of integrated efforts to address this issue.

## 4. Materials and Methods

### 4.1. Sample Collection, Microbiological Analysis, and Antimicrobial Sensitivity Assay (ASA)

Wastewater samples from different sites of Pakistan, including sewage, household wastewater, and canals of different sites, were collected over a two-year period from August 2020 to July 2022. Fifty ml of each water sample was collected in sterile falcon tubes, transported, and processed immediately at the Bacterial Pathogen Laboratory, Health Biotechnology Division, National Institute for Biotechnology and Genetic Engineering College, Pakistan Institute of Engineering and Applied Sciences (NIBGE-C, PIEAS) Faisalabad, Pakistan. Otherwise they were kept at 4 °C to be processed on the next day for isolation. The collected samples were enriched in buffered peptone water (BPW; Oxoid, UK) in a ratio of 1:9 (sample:BPW) and incubated at 37 °C for 18–24 h. One loop-full of the enriched culture was streaked onto CHROMagar™ mSuperCARBA™ plates (CHROMagar, Saint-Denis, France), which is a selective and differential chromogenic culture medium for the isolation and detection of CRB, and then incubated at 37 °C for 18–24 h. Based on the species-enrichment guidelines provided by CHROMagar™ mSuperCARBA™ media, the isolates were grouped into three categories; pink for suspected *E. coli*, metallic blue colonies for presumptive *Klebsiella*, *Enterobacter*, or *Citrobacter*, and cream, opaque colonies for suspected *Acinetobacter*. These colored colonies were further streaked on MacConkey agar (Oxoid: CM0007) and CHROMagar™ mSuperCARBA™ plates to purify the individual colonies. The pure presumptive *E. coli* isolates were further identified for the presence of the *uidA* gene that encodes a β-D-glucuronidase enzyme used for the identification of *E. coli*. PCR profile and conditions were followed as published previously [58].

Antibiograms of each of the isolates were detected using standard Kirby–Bauer disc diffusion protocol [59] and the results were interpreted using Clinical and Laboratory Standards Institute (CLSI) [60] guidelines and breakpoints. Briefly, the bacterial cultures were grown overnight in Tryptone Soya Broth (TSB; Oxoid: CM0129, UK), diluted to the equal turbidity of freshly prepared 0.5 MacFarland standard and spread as a lawn culture on Mueller–Hinton agar (MHA; Oxoid: CM0337). The *E. coli* ATCC 25922 strain was used as quality control for this assay. The antibiotic discs were dispensed on MHA using a 6-cartridge antimicrobial susceptibility disc dispenser (Oxoid, UK). The isolates were tested against a panel of 10 antimicrobial discs (Oxoid, UK), commonly used in human and veterinary medicine including imipenem (10 μg), meropenem (10 μg), ertapenem (10 μg), doripenem (10 μg), ceftazidime (30 μg), cefotaxime (5 μg), ciprofloxacin (5 μg), gentamicin (10 μg), trimethoprim/sulfamethoxazole (25 μg), and nalidixic acid (30 µg). The diameters of inhibition zones were determined, and the isolates were categorized as resistant, intermediate resistant, or susceptible using the CLSI guidelines. The isolates with intermediate resistant were considered as “resistant″ in the Section 2.

### 4.2. Whole-Genome Sequencing and Bioinformatics Analysis

Thirty-two isolates were selected for whole-genome sequencing based on the following criteria: (a) phenotypic resistance to carbapenem and cephalosporins; (b) samples collected from different provinces/locations of Pakistan; (c) susceptible isolates for the comparative study; and (d) based on the ARGs’ specific PCR results (unpublished data). A single colony of selected isolates was suspended in 1 mL of TSB at 37 °C overnight, and the genomic DNA was extracted using the GeneJET DNA purification kit (Thermo Fisher Scientific, Waltham, Massachusetts, USA). The DNA samples were visualized and confirmed on ethidium bromide-stained 2% agarose gel electrophoresis system. The genomic libraries were prepared and sequenced using a previously published protocol [61]. Briefly, the Nextera XT DNA library preparation kit (Illumina, San Diego, CA, USA) was used to generate paired-end libraries, followed by sequencing on an Illumina MiSeq (Illumina, San Diego, CA, USA) using the MiSeq Reagent V2 500 cycle kit (Illumina, USA). The representative *E. coli* isolate harboring the *bla* _NDM-5_ gene, 41EC2, was additionally sequenced using long-read sequencing approach on a Mk1C device (Oxford Nanopore Technologies, Oxford, UK) using Native Barcoding Kit 24 V14 (SQK-NBD114.24) with R10.4.1 flow cell. The bases were named using Guppy v6.4.6, and the assembly of Illumina and nanopore sequencing reads were performed using Unicycler v0.4.9b to ascertain the whole-genome and plasmid sequences for this isolate [62].

The raw reads were processed using the same protocol as published previously [34]. Briefly, the raw reads obtained were checked for quality using FastQC v0.12.1 and multiQC v1.19. The adapters and low-quality reads were removed to achieve a Phred score of 33 with Trimmomatic v0.39. The quality of trimmed reads was checked thorough FastQc and multiQC. SPAdes v3.15.5 was performed on the trimmed reads for de novo assembly of the genomes. The contigs with length and coverage less than 500 bp and 2X, respectively, were removed from the assembled genomes [63]. The quality of the assemblies was checked with QUAST v 3.9, while the contamination and completeness of the assembled genomes were evaluated with Checkm2 [64,65]. The bacterial isolates were identified using the following approaches: (a) the *k-mer* approach, using the Genomic Approximation Method for Bacterial Identification and Tracking tool (GAMBIT) [66]; (b), the average nucleotide identity analysis using FastANI v1.33, including all the species reference genome sequences available at NCBI RefSeq database for a genus (list of genomes with accessions available in Supplementary File Table S2) [67]; and (c) cross-checking of species confirmations with the Public databases for molecular typing and microbial genome diversity (BIGSdb) (https://pubmlst.org/bigsdb?db=pubmlst_rmlst_seqdef_kiosk). The assembled genomes were annotated by Prokka (Rapid Prokaryotic genome annotation, Prokka v1.14.6) [68]. The phylotypes and serotypes for *E. coli* isolates were determined using EzClermont and ABRicate 1.0.1 (https://github.com/tseemann/abricate) with echo database, respectively [69]. The sequence types (ST) for the available schemes were determined using the BIGSdb database (https://pubmlst.org/), and the assemblies were screened using the MLST tool (v2.23.0) and the online version of the PubMLST database [70] (https://github.com/tseemann/mlst). The Kaptive web tool was additionally used to determine the capsule types for the *Klebsiella* isolates (https://journals.asm.org/doi/10.1128/jcm.00197-18). The acquired ARGs and point mutations in the resistance genes were identified in ABRicate using CGE Resfinder database (https://bitbucket.org/genomicepidemiology/resfinder) and AMRFinderPlus v3.12.8 with database v2024-01-31.1, respectively [71]. 

The assemblies were searched for integrons using IntegronFinder (v2.0) with options [--local-max --func-annot –gbk] [72,73]. The gene cassettes from IntegronFinder’s GenBank-formatted output file were further validated and identified with Prokka, Resfinder and BLASTp. Additionally, sequence searches were performed using DIAMOND in eggNOG-mapper (v 2.1.12) to functionally annotate the gene cassettes based on the eggNOG orthology data [74]. Additionally, Plasmid Hunter was used to screen for integron-positive contigs and to classify them as plasmid- or chromosomal-borne contigs [75]. The plasmid replicons were predicted using PlasmidFinder in ABRicate (https://github.com/tseemann/abricate). The complete assembly of plasmid from 41EC2 was characterized, visualized, and compared to other similar plasmids using Mob-suite v1.4.9 and BLAST Ring Image Generator (BRIG) v.0.95, respectively [76,77]. Moreover, the plasmid multilocus sequence typing (pMLST) was performed using the pMLST tool (https://cge.food.dtu.dk/services/pMLST/).

## 5. Limitations of the Study

This study has some limitations. The bacterial isolates analyzed may not fully represent the overall resistance in wastewater, as only thirty-two isolates were selected from different locations. This limited sampling may be an underestimation or overestimation of CRB presence. Additionally, the large amount of debris and fungal growth also presents a challenge in obtaining single bacterial colonies, ultimately leading to the underestimation of ARB in the samples. Future studies are needed to assess the resistome content of bacterial isolates nationwide and by incorporating more samples collected at different time points of the year to provide a more comprehensive understanding of the resistome in wastewater.

## 6. Conclusions

For the first time, the data presented here highlight the role of environment samples as hotspots for WHO-priority bacterial pathogens and ARGs in Pakistan. The detection of clinically relevant ARGs, particularly the carbapenemases variants, *bla* _NDM_ and *bla* _OXA_, detected in this study, underscores the role of wastewater as a significant reservoir of carbapenmases. The presence of carbapenem- and colistin-resistant isolates, the last-resort antibiotics, suggests a large arsenal of mechanisms in these isolates under antimicrobial pressure and is a serious threat to public health and environmental integrity. Therefore, it is crucial to focus on community wastewater to prevent the dissemination of these key ARGs by novel genetic arrangements via mobile genetic elements, especially the integrons, or by horizontal gene transfer of *bla* _NDM-5_ on conjugative plasmids. Implementing effective preventive and control measures is essential to reducing the spread of these emerging threats. This research contributes valuable insights into the dynamics of antimicrobial resistance from a One Health perspective.

## Figures and Tables

**Table 1:** Phenotypic antimicrobial susceptibility profile of the sequenced isolates from this study.

Sample ID	Species	Antimicrobial Susceptibility Profile									
		β-lactams						Quinolones		Combination	Aminoglycosides
		Carbapenem				Cephalosporins					
		IPM	MEM	DOR	ETP	CTX	CAZ	CIP	NAL	SXT	GEN
19EC2	*Escherichia coli*	I	S	S	I	R	R	R	R	R	S
20EC1	*Escherichia coli*	R	R	R	R	R	R	R	R	R	R
21EC2	*Escherichia coli*	R	R	R	R	R	R	R	R	R	R
22EC1	*Escherichia coli*	R	R	R	R	R	R	R	R	I	I
22EC2	*Escherichia coli*	R	R	R	R	R	R	R	R	S	R
23EC1	*Escherichia coli*	R	R	R	R	R	R	R	R	S	I
23AC1	*Escherichia coli*	R	R	R	R	R	R	R	R	R	R
26AC1	*Escherichia coli*	R	R	R	R	R	R	R	R	R	I
30EC1	*Escherichia coli*	R	R	R	R	R	R	R	R	R	R
36AC2	*Escherichia coli*	R	R	R	R	R	R	R	R	R	I
37EC1	*Escherichia coli*	S	R	I	R	R	R	R	I	S	S
37EC2	*Escherichia coli*	R	R	R	R	R	R	R	R	R	R
41EC2	*Escherichia coli*	R	R	R	R	R	R	R	R	R	I
42AC1	*Escherichia coli*	S	S	S	R	R	S	S	S	S	S
43EC1	*Escherichia coli*	R	I	R	R	R	R	R	R	R	R
45EC1	*Escherichia coli*	R	R	R	R	R	R	R	R	R	I
68EC2	*Escherichia coli*	I	I	R	S	R	R	R	R	S	S
76EC1	*Escherichia coli*	I	S	S	S	R	I	I	I	R	S
EB7-1	*Klebsiella quasipneumoniae*	R	R	R	R	R	R	R	R	R	I
EC117	*Klebsiella pneumoniae*	I	I	I	I	R	S	R	I	I	S
EC118	*Klebsiella pneumoniae*	S	I	S	S	I	S	I	S	S	S
EC139	*Klebsiella pneumoniae*	S	I	I	I	R	I	R	S	S	S
22EB1	*Klebsiella pneumoniae*	R	R	R	R	R	R	R	R	S	I
EB1-3	*Citobacter telavivensis*	S	R	I	S	R	S	I	S	S	S
66EB1	*Citobacter portucalensis*	R	S	I	I	I	R	S	R	R	S
17EB1	*Raoultella ornithinolytica*	S	S	S	S	S	S	R	S	R	S
68EB1	*Raoultella planticola*	I	I	I	R	R	R	I	I	R	R
EC145	*Enterobacter cloacae*	R	S	I	I	R	I	R	S	S	R
51AC1	*Acinetobacter baumannii*	R	R	R	R	R	I	R	S	I	S
77AC2	*Morganella morganii*	R	I	R	S	R	R	R	R	R	R
78EB1	*Serratia ureilytica*	R	I	R	S	R	I	S	S	S	S
78EB2	*Serratia nevei*	I	S	S	S	R	I	I	S	S	S

Definitions: IPM: imipenem; MEM: meropenem DOR: doripenem: ETP: ertapenem; CTX: cefotaxime; CAZ: ceftazidime; CIP: ciprofloxacin; NAL: nalidixic Acid; SXT: trimethoprim-sulfamethoxazole; GEN: gentamycin; abbreviation of standard antibiotic codes from (https://journals.asm.org/abbreviations-conventions, accessed on 6 June 2024). Resistant: R, I: intermediate, S: susceptible.

**Table 2:** Correlation between phenotypic resistance profiles and the genetic mechanisms detected in the isolates sequenced in this study.

Sample ID	Species	βlactam				Quinolones		Combination		Aminoglycosides		No Phenotypes Tested
		Carbapenem		Cephalosporins								
		Phenotype	Genetic Mechanism	Phenotype	Genetic Mechanism	Phenotype	Genetic Mechanism	Phenotype	Genetic Mechanism	Phenotype	Genetic Mechanism	Other Genetic Mechanism
19EC2	*Escherichia coli*	I (IPM, ETP), S (MEM, DOR)	fstI I336IKYR1	R	*OXA-1*, *CTX-M-15*, *CMY-16*	R	*aac(6′)-Ib-cr_1*, *gyrA* (S83L and D837N), *parC* (S80I and S57T), *parE* (S458A)	R	*sul2*, *dfrA1*	S	*aph(3″)-Ib_2*, *aph(6)-Id_1*	*tet(A)*, glpT_E448K
20EC1	*Escherichia coli*	R	*NDM-5*, fstI N337NYRIN	R	*OXA-1*, *CTX-M-15*, *TEM-1B*	R	*aac(6′)-Ib-cr_1*, *gyrA* (S83L and D837N), *parC* (S80I), *parE* (S458A)	R	*sul1*, *dfrA17*	R	*aadA5*, *aac(3)-IIa*	*catA1*, *mph(A)*, *tet(B)*, *ble*, *qacEdelta1*, glpT_E448K, cyanA_S352T
21EC2	*Escherichia coli*	R	*NDM-5*, fstI I336IKYR1	R	*OXA-1*, *CTX-M-15*	R	*aac(6′)-Ib-cr_1*, *gyrA* (S83L and D837N), *parC* (S80I), *parE* (S458A)	R	*sul1*, *dfrA17*	R	*aac(6′)-Ib-cr_1*, *aadA5*, *aac(3)-IIa*	*mph(A)*, *ble*, *qacEdelta1*, glpT_E448K
22EC1	*Escherichia coli*	R	*OXA-181*	R	*CTX-M-15*, *TEM-35*, blaTEMp_C32T	R	*qnrS1*	I		I		glpT_E448K
22EC2	*Escherichia coli*	R	Nil	R	*CTX-M-15*, *TEM-35*, blaTEMp_C32T	R	*qnrS1*	S		R		glpT_E448K
23EC1	*Escherichia coli*	R	Nil	R	*TEM-1B*	R	*gyrA* (S83L and D837N), *parC* (S80I), *parE* (S458A)	S	Nil	I	Nil	*mph(A)*, pmrB_Y358N, glpT_E448K
23AC1	*Escherichia coli*	R	*OXA-181*, fstI N337NYRIN	R	*OXA-1*, *CTX-M-15*, *CMY-2*	R	*qnrS1*, *aac(6′)-Ib-cr_1*, *gyrA* (S83L and D837N), *parC* (S80I), *parE* (S458A), *parE* (S458A)	R	*sul1*, *sul2*	R	*aac(6′)-Ib-cr_1*, *aadA5*, *aac(3)-IId*, *aph(3″)-Ib_5*, *aph(6)-Id*	*mph(A)*, *tet(B)*, *qacEdelta1*, pmrB_Y358N, glpT_E448K
26AC1	*Escherichia coli*	R	*OXA-181*, fstI I336IKYR1	R	*CTX-M-15*	R	*qnrS1*, *gyrA* (S83L and D837N), *parC* (S80I), *parE* (S458A)	R	*sul1*, *dfrA17*	I	*aadA5*,	*mph(A)*, *qacEdelta1*, pmrB_Y358N, glpT_E448K
30EC1	*Escherichia coli*	R	*NDM-5*, fstI N337NYRIN	R	*OXA-1*	R	*gyrA* (S83L and D837N), *parC* (S80I), *parE* (S458A)	R	*sul1*, *dfrA12*	R	*aadA2*,	glpT_E448K
36AC2	*Escherichia coli*	R	*OXA-181*, fstI I336IKYR1	R	*CTX-M-15*, *TEM-169*	R	*qnrS1*, *gyrA* (S83L and D837N), *parC* (S80I), *parE* (S458A)	R	*sul1*, *dfrA17*	I	*aadA5*,	*mph(A)*, *qacEdelta1*, pmrB_Y358N, glpT_E448K
37EC1	*Escherichia coli*	R (MEM, ETP), I (DOR), S (IMP)	*OXA-181*	R	*CTX-M-15*, *TEM-35*, blaTEMp_C32T	R (CIP), I (NAL)	*qnrS1*	S	Nil	S	Nil	*mph(A)*, glpT_E448K
37EC2	*Escherichia coli*	R	*NDM-5*, fstI I336IKYR1	R	*OXA-1*, *CTX-M-15*	R	*aac(6′)-Ib-cr_1*, *gyrA* (S83L and D837N), *parC* (S80I), *parE* (S458A)	R	*sul1*, *dfrA12*, *dfrA17*	R	*aadA2*, *aadA5*, *aac(3)-IIa*	*mph(A)*, *tet(B*, *ble*, *qacEdelta1*, glpT_E448K, cyanA_S352T
41EC2	*Escherichia coli*	R	*NDM-5*, fstI N337NYRIN	R	*OXA-1*, *CTX-M-15*, *TEM-1B*	R	*aac(6′)-Ib-cr_1*, *gyrA* (S83L and D837N), *parC* (S80I), *parE* (S458A)	R	*sul1*, *dfrA12*	I	*aadA2*, *aac(6′)-Ib-cr_1*	*mph(A)*, *tet(A)*, *ble*, *qacEdelta1*,
42AC1	*Escherichia coli*	R (ETP), S (IPM, MEM, DOR)	*OXA-181*, fstI I336IKYR1	R (CTX), S (CAZ)	*CTX-M-15*, *TEM-169*	S	*qnrS1*, *gyrA* (S83L and D837N), *parC* (S80I), *parE* (S458A)	S	*sul1*	S	*aadA5*	*mph(A)*, *qacEdelta1*, pmrB_Y358N, glpT_E448K
43EC1	*Escherichia coli*	R (IPM, DOR, ETP), I (MEM)	fstI N337NYRIN	R	*TEM-35*	R	*gyrA* (S83L and D837N), *parC* (S80I and E84G)	R	*sul2*, *dfrA1*	R	*aph(3″)-Ib_5*	*mph(A)*, *tet(B)*,
45EC1	*Escherichia coli*	R	*NDM-5*, fstI I336IKYR1	R	*CMY-16*	R	*gyrA* (S83L and D837N), *parC* (S80I)	R	*sul1*, *dfrA1*, *dfrA12*	I	*aadA2*,	*mph(A)*, *tet(A)*, *ble*, *qacEdelta1*, pmrB_Y358N, glpT_E448K
68EC2	*Escherichia coli*	R (DOR), I (IPM, MEM), S (ETP)	Nil	R	*SHV-12*	R	*qnrS1*	S	Nil	S	Nil	glpT_E448K
76EC1	*Escherichia coli*	I (IPM), S (ETP, MEM, DOR)	Nil	R (CTX), I (CAZ)	*CTX-M-15*, *TEM-1B*	I	*qnrS1*	R	*sul2*, *dfrA14*	S	*aph(3″)-Ib_5*, *aph(6)-Id*	*tet(A)*
EB7-1	*Klebsiella quasipneumoniae*	R	Nil	R	*OKP-B-15*, *OXA-1*, *CTX-M-15*, *TEM-1B*	R	*qnrB1*, *aac(6′)-Ib-cr5*, *oqxA/B*	R	*sul2*, *dfrA14*	I	*aph(3″)-Ib_5*, *aph(6)-Id*	*fosA_6*, *tet(A)*
EC117	*Klebsiella pneumoniae*	I	Nil	R (CTX), I (CAZ)	*SHV-187*	R (CIP), I (NAL)	*qnrS1*, *oqxA/B*	I	*dfrA14*	S	Nil	*fosA_3*
EC118	*Klebsiella pneumoniae*	I (MEM), S (IPM, ETP, DOR)	Nil	I (CTX), S (CAZ)	*SHV-187*	I (CIP), S (NAL)	*oqxA/B*	S	Nil	S	Nil	*fosA_3*
EC139	*Klebsiella pneumoniae*	I (MEM, ETP, DOR), S (IPM)	Nil	R (CTX), I (CAZ)	*SHV-187*	R (CIP), S (NAL)	*oqxA/B*	S	Nil	S	Nil	*fosA_3*
22EB1	*Klebsiella pneumoniae*	R	NDM-1	I	*OXA-9*, *CTX-M-15*, *DHA-1*, *SHV-145*, *TEM-1A*	R	*qnrS1*, *oqxA/B*	S	Nil	I	*aadA1*, *aac(6′)-Ib*, *aph(3″)-Ib_2*, *aph(3′)-VI*, *aph(6)-Id*	*ble*, *fosA6*
EB1-3	*Citobacter telavivensis*	R(MEM), I(DOR), S (IPM, ETP)	Nil	R (CTX), I (CAZ)	*SED1*	I (CIP), S (NAL)	*oqxA/B*	S	Nil	S	Nil	Nil
66EB1	*Citobacter portucalensis*	R (IPM), I (ETP, DOR), S (MEM)	Nil	R (CAZ), I (CTX)	*CMY-119*, *DHA-1*	S (CIP), R (NAL)	*qnrB43*, *qnrB4*	R	*Sul1*, *dfrA7*	S	Nil	*mph(A)*, *tet(A)*
17EB1	*Raoultella ornithinolytica*	S	Nil	S	*LAP-2*, *PLA-1a*	R (CIP), S (NAL)	*qnrS1*	R	*Sul1*, *dfrA1*	S	Nil	*fosA_2*, *tet(B)*, *qacEdelta1*
68EB1	*Raoultella planticola*	R (ETP), I (IMP, MEM, DOR)	Nil	R	*CTX-M-15*, *PLA-5A*, *TEM-1A*	I	*qnrB1*, *oqxB*	R	*Sul2*, *dfrA14*	R	*aac(3)-IId*, *aph(3″)-Ib_5*, *aph(6)-Id*	*fosA_2*
EC145	*Enterobacter cloacae*	R (IPM), I (ETP, DOR), S (MEM)	Nil	R (CTX), I (CAZ)	*CMH_3*	R (CIP), S (NAL)	Nil	S	Nil	R	Nil	Nil
51AC1	*Acinetobacter baumannii*	R	OXA-72	R (CTX), I (CAZ)	*OXA-70*, *ADC_25*	R (CIP), S (NAL)	Nil	I	Nil	S	*ant(3″)_IIa*	Nil
77AC2	*Morganella morganii*	R (IMP, DOR), I (MEM), S (ETP)	Nil	R	*DHA-13*	R	Nil	R	*sul1*, *dfrA10*	R	*ant(2″)_Ia*, *aph(3′)-Ia*	*catA1*, *tet(B)*, *qacEdelta1*
78EB1	*Serratia ureilytica*	R (IMP, DOR), I (MEM), S (ETP)	Nil	R (CTX), I (CAZ)	*SST-1*	S	Nil	S	Nil	S	*aac(6′)-Ic*	
78EB2	*Serratia nevei*	I (IMP), S (MEM, ETP, DOR)	Nil	R (CTX), I (CAZ)	*SRT-1*	I (CIP), S (NAL)	Nil	S	Nil	S	*aac(6′)-Ic*	Nil

**Table 3:** Detection of integron elements and their associated gene cassettes in the wastewater isolates using IntegronFinder 2.0.

Sample ID	Species	Contig Origin	Size of Integron (bp)	Gene Cassette(s) and Order
20EC1	*Escherichia coli*	P	2802	*dfrA17-aadA5-qacEΔ1*
21EC2	*Escherichia coli*	P	2802	*dfrA17- aadA5-qacEΔ1*
30EC1	*Escherichia coli*	P	3366	*dfrA12-aadA2-qacEΔ1*
41EC2	*Escherichia coli*	P	3240	*dfrA12-aadA2-qacEΔ1*
45EC1	*Escherichia coli*	P	3240	*dfrA12-aadA2-qacEΔ1*
76EC1	*Escherichia coli*	P	1952	*dfrA14-Tnp(*partial*)*
EB7-1	*Klebsiella quasipneumoniae*	P	2130	*dfrA14-mobC*
EC117	*Klebsiella pneumoniae*	P	2130	*dfrA14-mobC*
66EB1	*Citobacter portucalensis*	C	1691	*dfrA7-qacEΔ1*
68EB1	*Raoultella planticola*	P	1723	*dfrA14*
77AC2	*Morganella morganii*	P	2200	*ant(2″)-Ia- qacEΔ1*

P = Plasmid, C = Chromosome.

## Data Availability

The raw reads for all the studied isolates are available under Bio Project PRJNA1106557 and the list of NCBI accessions for these isolates are included in Supplementary file S1. Complete closed genome sequences for 41EC2 analyzed for this study are deposited in NCBI, including chromosome (Accession No. CP1557270) and plasmid (Accession No. CP155728), under the same Bio Project number (PRJNA1106557).

## Supplementary Materials

The following supporting information can be downloaded at: https://www.mdpi.com/article/10.3390/antibiotics13100937/s1, Table S1: Summary of isolates analyzed and submitted under Bio Project PRJNA1106557; Table S2: The list of reference genome names and NCBI Ref Seq accession used for ANI calculations; Table S3: MLST and Capsular typing of the *Klebsiella pneumonia* isolates from this study; Table S4: Sequences producing significant alignments with *bla* _NDM-1_ from different species, sources, and geographic locations.
